# High Preoperative Serum Syndecan-1, a Marker of Endothelial Glycocalyx Degradation, and Severe Acute Kidney Injury after Valvular Heart Surgery

**DOI:** 10.3390/jcm9061803

**Published:** 2020-06-10

**Authors:** Hye-Bin Kim, Sarah Soh, Young-Lan Kwak, Jae Chan Bae, Sang Hwa Kang, Jong Wook Song

**Affiliations:** 1 Department of Anesthesiology and Pain Medicine, Anesthesia and Pain Research Institute, Yonsei University College of Medicine, Seoul 03722, Korea; kakddugi@yuhs.ac (H.-B.K.); yeonchoo@yuhs.ac (S.S.); ylkwak@yuhs.ac (Y.-L.K.); 2 Department of Anesthesiology and Pain Medicine, Yonsei University College of Medicine, Seoul 03722, Korea; bjchn89@yuhs.ac; 3 Department of Anesthesiology and Pain Medicine, National Health Insurance Service Ilsan Hospital, Goyang 10444, Korea; moong73@nhimc.or.kr

**Keywords:** acute kidney injury, cardiac surgical procedures, endothelium, glycocalyx, syndecan-1

## Abstract

Degradation of endothelial glycocalyx (EG) is associated with inflammation and endothelial dysfunction, which may contribute to the development of acute kidney injury (AKI). We investigated the association between a marker of EG degradation and AKI after valvular heart surgery. Serum syndecan-1 concentrations were measured at induction of anesthesia and discontinuation of cardiopulmonary bypass in 250 patients. Severe AKI was defined as Kidney Disease: Improving Global Outcomes Criteria Stage 2 or 3. Severe AKI occurred in 13 patients (5%). Receiver operating characteristic analysis of preoperative syndecan-1 to predict severe AKI showed area under curve of 0.714 (95% confidence interval (CI), 0.575–0.853; *p* = 0.009). The optimal cut-off value was 90 ng/mL, with a sensitivity of 61.5% and specificity of 78.5%. In multivariable analysis, both preoperative syndecan-1 ≥ 90 ng/mL and Cleveland Clinic Foundation score independently predicted severe AKI. Severe tricuspid regurgitation was more frequent (42.4% vs. 17.8%, *p* < 0.001), and baseline right ventricular systolic pressure (41 (33–51) mmHg vs. 33 (27–43) mmHg, *p* = 0.001) and TNF-α (1.85 (1.37–2.43) pg/mL vs. 1.45 (1.14–1.92) pg/mL, *p* <0.001) were higher in patients with high preoperative syndecan-1. Patients with high preoperative syndecan-1 had longer hospital stay (16 (12–24) days vs. 13 (11–17) days, *p* = 0.001). In conclusion, a high preoperative syndecan-1 concentration greater than 90 ng/mL was able to predict severe AKI after valvular heart surgery and was associated with prolonged hospitalization.

## 1. Introduction

Acute kidney injury (AKI) is a common complication after cardiac surgery, with an incidence of 5–42% depending on the definition of AKI, extent of the procedure, and patients’ characteristics [1]. AKI after cardiac surgery, including a modest elevation in serum creatinine, is independently associated with increased mortality and morbidity [2]. However, the prognosis of severe AKI is even worse, as mortality increases sharply with more advanced stages of AKI [3]. Furthermore, AKI severity is associated with incremental risk of progression to chronic kidney disease [4]. As there are no proven remedies to treat AKI, strategies to prevent it and alleviate its severity are crucial. Preoperative risk stratification and early detection of injury may aid in the early implementation of such strategies and optimization of patient care. 

Endothelial glycocalyx (EG) is a gel-like structure covering the luminal surface of the endothelium. It consists of various glycosaminoglycans and associated plasma membrane-bound proteoglycans [5]. EG is involved in essential endothelial function and vascular homeostasis, including the regulation of vascular permeability, mechanotransduction of blood flow-induced shear stress, and interactions between leukocytes and endothelium [6]. EG is degraded under oxidative stress, ischemia-reperfusion, and inflammation, resulting in increased vascular permeability and interstitial edema, an exaggerated inflammatory response involving the upregulation of various cell membrane adhesion molecules and leukocyte-endothelial cell interactions [7,8]. These changes are implicated in the development of AKI in the context of endothelial dysfunction [9]. 

EG degradation has been observed in critically ill patients and is associated with poor outcomes [10,11,12,13]. It has also been observed in cardiac and major vascular surgery as a perioperative increase in serum concentrations of EG constituents, e.g., syndecan-1 [14,15,16,17,18]. However, evidence regarding its impact on perioperative outcomes of cardiac surgical patients is limited. Because the conditions that induce EG breakdown and its pathophysiologic consequences are closely related to cardiac surgery-associated AKI, we hypothesized that perioperative EG degradation may be associated with AKI development after valvular heart surgery. Thus, we aimed to investigate the association between syndecan-1, a marker of EG degradation, and AKI in patients undergoing valvular heart surgery.

## 2. Materials and Methods

### 2.1. Participants

This prospective, observational study was conducted at Severance Cardiovascular Hospital, Yonsei University Health System, Seoul, Republic of Korea, approved by our institutional review board (IRB no.: 4-2017-0340) and registered at ClinicalTrials.gov (NCT03197051) before recruitment started. A total of 250 patients older than 20 years scheduled for elective valvular heart surgery between June 2017 and July 2018 were enrolled after obtaining written informed consent. Exclusion criteria were minimally invasive surgery, chronic kidney disease stage ≥ 4 (estimated glomerular filtration rate < 30 mL/min/1.73 m^2^) or preoperative AKI, infective endocarditis, malignancies, or preoperative mechanical circulatory support. 

### 2.2. Protocol

All patients received standardized perioperative management according to institutional guidelines. Anesthesia was induced by intravenous midazolam (0.05–0.07 mg/kg) and sufentanil (1.5–2 μg/kg) and maintained with inhaled sevoflurane and continuous infusion of sufentanil. Heparin was administered at an initial dosage of 300 IU/kg to achieve an activated clotting time above 480 s. The cardiopulmonary bypass (CPB) circuit was primed with a 1.6-L priming solution consisting of 100 mL of 20% albumin, 20% mannitol (0.5 g/kg), sodium bicarbonate (20 mEq), heparin (2000 IU), and plasmalyte (Plasma Solution A Inj.; CJ Pharma, Seoul, Korea). A non-pulsatile pump flow rate was maintained at 2.0–2.4 L/min/m^2^ under mild hypothermia (32–33 °C) using alpha-stat management. After weaning from CPB, heparin was antagonized by protamine sulphate (3 mg/kg). All patients were transferred to the intensive care unit (ICU) postoperatively.

Norepinephrine (up to 0.5 μg/kg/min) and vasopressin (up to 4 IU/h) were used as primary and second-line vasopressors, respectively. Milrinone was administered to treat ventricular systolic dysfunction. Hematocrit levels were maintained above 20% during CPB and 25% before and after CPB with the transfusion of packed red blood cells and ultrafiltration/reinfusion of intraoperatively salvaged blood during CPB. For fluid resuscitation, plasmalyte and synthetic balanced colloid solution (6% hydroxyethyl starch 130/0.4, Volulyte; Fresenius Kabi, Seoul, Korea; up to a maximal dose of 20 mL/kg/d) were used. Allogeneic plasma products including fresh frozen plasma, platelet concentrate, and cryoprecipitate were transfused at the discretion of the attending physician based on thromboelastography, prothrombin time, and platelet count.

### 2.3. Measurements

Arterial blood samples were taken at the induction of anesthesia and after the reversal of heparinization with protamine, centrifuged at 1000 × *g* for 15 min, and stored at −80 °C for subsequent analysis. Enzyme-linked immunosorbent assay was used to measure the serum concentrations of syndecan-1 (Abcam, Cambridge, MA, USA, Cat No. ab46506, sensitivity: 4.94 ng/mL), heparan sulfate (MyBioSource, San Diego, CA, USA, MBS267373), tumor necrosis factor-α (TNF-α; R&D Systems, Minneapolis, MN, USA, HSTA00E), and interleukin-6 (IL-6; R&D Systems, HS600B). 

Serum creatinine concentrations were assessed 1 day before surgery and postoperatively at 6, 24, and 48 h. Additional measurements were performed at the discretion of the attending physician, with values measured within 7 postoperative days used for the diagnosis and staging of AKI. C-reactive protein (CRP) and hematologic variables including leukocyte count, hematocrit, and platelet count were assessed 1 day before surgery and postoperatively at 6, 24, and 48 h. Perioperative fluid balance, transfusion requirements, and vasopressor and inotropic drug requirements were recorded for 48 h postoperatively. Hemodynamic parameters, including mean arterial pressure, cardiac index, heart rate, central venous pressure (CVP), mean pulmonary arterial pressure, and mixed venous oxygen saturation, were recorded after induction of anesthesia, at the discontinuation of CPB, and postoperatively at 6 and 24 h.

Postoperative 30-day morbidity end points, including mortality, permanent stroke, reoperation due to bleeding, deep sternal wound infection, prolonged mechanical ventilation > 24 h, and length of intensive care unit (ICU) and hospital stays, were also assessed.

The primary endpoint was the association between perioperative serum syndecan-1 concentration, a marker of EG degradation, and the incidence of AKI after valvular heart surgery. Kidney Disease: Improving Global Outcomes (KDIGO) criteria was used to define AKI (Stages 1–3) and severe AKI (Stages 2–3), as follows: Stage 1, 1.5–1.9 times baseline or ≥ 0.3 mg/dL increase in serum creatinine or urine output < 0.5 mL/kg/h for 6–12 h; Stage 2, serum creatinine 2.0–2.9 times baseline or urine output < 0.5 mL/kg/h for ≥ 12 h; Stage 3, serum creatinine 3.0 times baseline or increase in serum creatinine to ≥ 4.0 mg/dL or initiation of renal replacement therapy or urine output <0.3 mL/kg/h for ≥24 h or anuria for ≥12 h.

### 2.4. Statistical Analysis

We assumed that the incidence of severe AKI would be 10% and a detectable odds ratio (OR) corresponding to an increase of one standard deviation from the mean value of syndecan-1 would be 2.0. With a Type I error of 0.05 and power of 95%, 237 subjects were required. Considering the dropout rate, we decided to enroll 250 patients.

Continuous variables are shown as mean (standard deviation) or median with interquartile range (25th–75th percentile). Categorical variables are expressed as number of patients and percentages. The association between serum syndecan-1 and severe AKI was analyzed by logistic regression analysis, and the optimal cut-off value for predicting severe AKI was assessed by receiver operating characteristic (ROC) analysis. Multivariable analysis was performed by considering the serum syndecan-1 concentration along with the Cleveland Clinic Foundation score, which is a previously acknowledged clinical prediction model for acute renal failure after cardiac surgery [19]. Continuous variables were compared using independent Student’s t or Mann–Whitney U tests depending on the results of Shapiro–Wilk tests for normality, and categorical variables were compared using chi-square or Fisher’s exact tests as appropriate. Repeated measures analysis of variance (ANOVA) or Friedman’s test was used to analyze repeatedly measured variables, then *p* values of post hoc tests were adjusted with the Bonferroni’s method. SPSS version 23.0 (SPSS Inc., Chicago, IL, USA) and NCSS 12 (NCSS LLC, Kaysville, UT, USA) were used for analysis. Differences with *p* < 0.05 were considered statistically significant except comparisons between high vs. low syndecan-1 groups, in which *p* < 0.01 were considered statistically significant.

## 3. Results

### 3.1. Prediction of Postoperative Severe AKI

**Figure 1:**
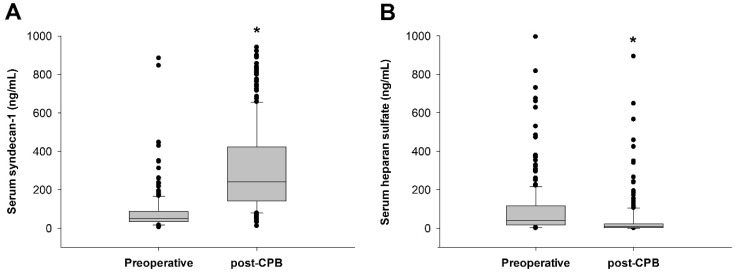
Serum concentrations of syndecan-1 (**A**) and heparan sulfate (**B**). The samples were drawn at the induction of anesthesia (preoperative) and after the reversal of heparinization with protamine (post-cardiopulmonary bypass (CPB)). Syndecan-1 was increased from its preoperative value whereas heparan sulfate decreased; * *p* < 0.001 vs. preoperative concentration. Abbreviation: CPB, cardiopulmonary bypass.

**Figure 2:**
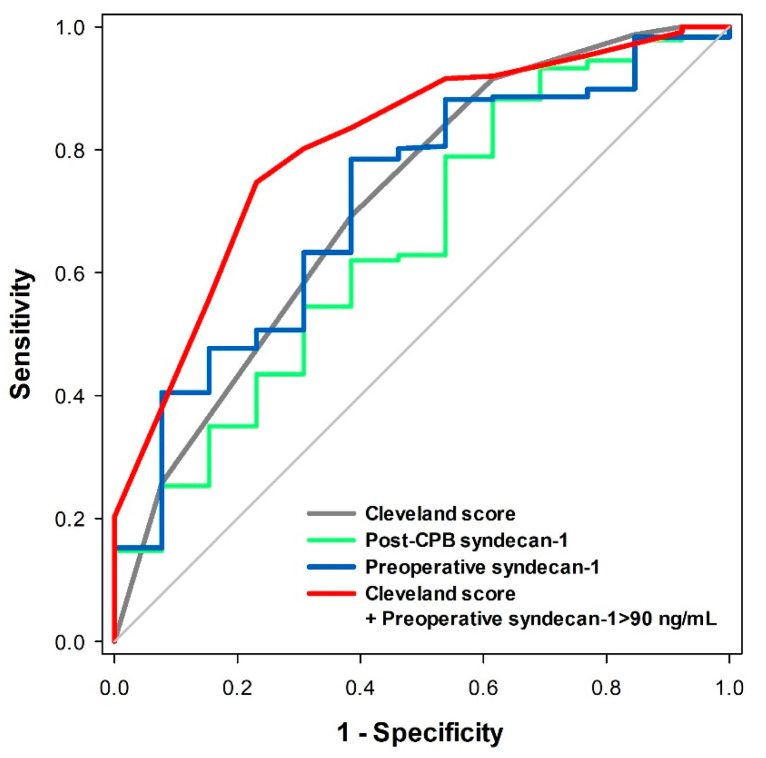
Receiver operating characteristic (ROC) curves for severe acute kidney injury (AKI) with each potential predictors. Blue line: preoperative syndecan-1 concentration (area under the ROC curve (AUC), 0.714; 95% CI, 0.575–0.853; *p* = 0.009); green line: post-CPB syndecan-1 concentration (AUC, 0.653; 95% CI, 0.497–0.809; *p* = 0.063); grey line: Cleveland Clinic Foundation score (AUC, 0.714; 95% CI, 0.560–0.869; *p* = 0.009); red line: Cleveland Clinic Foundation score + preoperative syndecan-1 concentration ≥ 90 ng/mL (AUC, 0.807; 95% CI, 0.690–0.924; *p* < 0.001). Abbreviations: AKI, acute kidney injury; AUC, area under the ROC curve; CI, confidence interval; CPB, cardiopulmonary bypass.

AKI and severe AKI occurred in 47 (18.8%) and 13 (5.2%) patients, respectively. The median serum syndecan-1 concentration was 51 (34–88) ng/mL at induction and increased to 241 (140–423) ng/mL after CPB (*p* < 0.001; Figure 1A). The median serum heparan sulfate concentration was 39.1 (16.1–116.1) ng/mL at induction and decreased to 8.3 (1.5–21.6) ng/mL after CPB (*p* < 0.001); Figure 1B). In univariable logistic regression, neither preoperative nor post-CPB syndecan-1 concentration was associated with the incidence of AKI (preoperative; OR, 1.002; 95% confidence interval (CI), 0.999–1.004; *p* = 0.290, post-CPB; OR, 1.000; 95% CI, 0.557–1.392; *p* = 0.587). Preoperative syndecan-1 concentration showed a non-significant association with the incidence of severe AKI (OR, 1.003; 95% confidence interval (CI), 1.000–1.006; *p* = 0.065), and post-CPB syndecan-1 concentration was significantly associated with the incidence of severe AKI (OR, 1.002; 95% CI, 1.000–1.005; *p* = 0.024). The fold increase of syndecan-1 was not associated with the incidence of severe AKI (OR, 0.934; 95% CI, 0.815–1.070; *p* = 0.323). The areas under the ROC curve (AUCs) of the preoperative and post-CPB syndecan-1 concentrations to predict severe AKI were 0.714 (95% CI, 0.575–0.853; *p* = 0.009) and 0.653 (95% CI, 0.497–0.809; *p* = 0.063), respectively (Figure 2). The optimal cut-off value of preoperative syndecan-1 for the prediction of severe AKI was 90 ng/mL, with a sensitivity of 61.5% and specificity of 78.5%. The Cleveland Clinic Foundation score for the prediction of acute renal failure after cardiac surgery [19] had an AUC of 0.714 (95% CI, 0.560–0.869; *p* = 0.009) in this study population (Figure 2). Multivariable logistic regression showed that both preoperative syndecan-1 concentration greater than 90 ng/mL (OR, 4.231; 95% CI, 1.249–14.33; *p* = 0.020) and Cleveland Clinic Foundation score (OR, 2.011; 95% CI, 1.202–3.364; *p* = 0.008) were independent predictors of severe AKI. Inclusion of these two variables in the prediction model yielded an AUC of 0.807 (95% CI, 0.690–0.924; *p* < 0.001), which was higher than the AUC of the Cleveland Clinic Foundation score alone, but not significantly so (*p* = 0.181; Figure 2). The preoperative syndecan-1 greater than 90 ng/mL also remained an independent predictor (OR, 4.650; 95% CI, 1.402–15.43; *p* = 0.012) after multivariable logistic regression analysis with the preoperative serum creatinine (OR, 6.951; 95% CI, 0.833–58.27; *p* = 0.080).

### 3.2. Comparison Between Low and High Preoperative Syndecan-1 Groups

According to ROC analysis, patients were divided into low (<90 ng/mL) and high (≥90 ng/mL) preoperative syndecan-1 groups. The preoperative characteristics of patients with low and high preoperative syndecan-1 concentrations are shown in Table 1. The proportion of patients with elevated preoperative serum creatinine was higher in the high syndecan-1 group with a borderline statistical significance (13.6% vs. 3.7%, *p* = 0.010). In the high syndecan-1 group, severe tricuspid regurgitation (42.4% vs. 17.8%, *p* < 0.001) was more frequent when compared with the low syndecan-1 group. Patients with high preoperative syndecan-1 had greater preoperative right ventricular systolic pressure than those with low syndecan-1 (41 (33–51) mmHg vs. 33 (27–43) mmHg, *p* = 0.001).

The overall incidence of AKI was significantly higher in patients with high preoperative syndecan-1 (32.2% vs. 14.7%, *p* = 0.003). The requirement for postoperative renal replacement therapy was 5.1% vs. 0.0%, high vs. low preoperative syndecan-1, respectively (*p* = 0.013, Table 2), and the incidence of oliguria was 6.8% vs. 1.5%, high vs. low preoperative syndecan-1, respectively (*p* = 0.025; Table 2). Changes in serum creatinine concentrations throughout the study period were comparable between groups (*p* = 0.621; Table 2). 

Patients with high preoperative syndecan-1 received more crystalloid fluid (6406 (5248–7554) mL vs. 5791 (4703–5791) mL, *p* = 0.010) and allogeneic plasma product (260 (0–1280)) mL vs. 0 (0–260) mL, *p* < 0.001), and had greater urine output (7000 (6165–7650) mL vs. 6175 (5340–7230) mL, *p* = 0.007), whereas synthetic colloid fluid administration, red blood cell transfusion, vasopressor and inotrope requirements, were not different between the two groups during the perioperative 48 h (Table 3). 

Patients with high preoperative syndecan-1 also showed higher syndecan-1 concentrations post-CPB than those with low preoperative syndecan-1 (383 (293–617) ng/mL vs. 198 (120–363) ng/mL, *p* < 0.001). Preoperative serum concentrations of TNF-α (1.85 (1.37–2.43) pg/mL vs. 1.45 (1.14–1.92) pg/mL, *p* <0.001) was higher in patients with high preoperative syndecan-1 (Table 4). Hematologic variables including hematocrit and leukocyte and platelet counts were similar between the groups, except the lower preoperative leukocyte count (5228 ± 2061 /μL vs. 6212 ± 1773 /μL, *p* = 0.004) and platelet count (178 ± 64 × 10^3^/μL vs. 195 ± 63 × 10^3^/μL, *p* = 0.004) in patients with high preoperative syndecan-1 (Supplementary Table S1). Hemodynamic variables including mean arterial pressure, heart rate, central venous pressure, mean pulmonary arterial pressure and cardiac index were also not different between the groups (Supplementary Table S2). 

Patients with high preoperative syndecan-1 had longer hospital stay (16 (12–24) days vs. 13 (11–17) days, *p* = 0.001) when compared with those with low syndecan-1 (Table 5). Postoperative outcomes including stroke, sternal infections, hemostatic reoperation, mechanical ventilation >24 h, and mortality were comparable between groups (Table 5). 

## 4. Discussion

In this observational study, preoperative syndecan-1 concentration greater than 90 ng/mL predicted severe AKI after valvular heart surgery, independently of a previously acknowledged clinical scoring system. Severe tricuspid regurgitation was more frequent, and baseline right ventricular systolic pressure was significantly greater in patients with high preoperative syndecan-1. Baseline serum inflammatory cytokine TNF-α concentration was also elevated in patients with high preoperative syndecan-1. Postoperatively, preoperative syndecan-1 greater than 90 ng/mL was associated with longer duration of hospital stay. 

The pathogenesis of AKI in association with cardiac surgery involves a complex interplay of direct damage, hemodynamics, ischemia-reperfusion, and inflammation [1]. Endothelial damage plays a central role in the development of AKI, leading to disruption of the permeability of the glomerulus and peritubular capillaries, facilitating leukocyte adhesion and migration, intravascular coagulation, platelet aggregation, loss of vasomotor autoregulation, and reduced perfusion [9]. Therefore, the conditions that induce EG degradation and its pathophysiological consequences are closely related to endothelial dysfunction in AKI. Indeed, endothelial injury characterized by EG disruption has been demonstrated in a swine model of CPB-induced AKI [20]. Thus, we initially hypothesized that increased serum markers of EG degradation may be associated with AKI after cardiac surgery.

Although the perioperative degradation of EG in cardiac surgery has been established in a number of studies [14,15,16,17,18], few have investigated its prognostic implications in the context of cardiac surgery-associated AKI. In a study by de Melo Bezerra Cavalcante and colleagues [21], elevated syndecan-1 concentrations in blood collected within 2 h postoperatively were independently associated with KDIGO Stage 2 or 3 AKI in patients undergoing pediatric cardiac surgery. They also showed that addition of syndecan-1 to their clinical AKI risk prediction model, which included age, sex, preoperative renal function, surgical complexity, and use and duration of CPB, improved the predictive performance of the model. However, they did not assess preoperative syndecan-1 concentrations, which may naturally influence its post-CPB concentration considering that it also reflects the degree of inflammatory tone [7,8]. In agreement with that study [21], post-CPB syndecan-1 concentrations were associated with KDIGO Stage 2 or 3 AKI in our study; however, only preoperative syndecan-1 exhibited significant predictive power in the ROC analysis. The fold increase in syndecan-1 also showed no association with AKI. The preoperative and post-CPB syndecan-1 concentrations showed significant correlation (Spearman’s ρ, 0.442; 95% CI, 0.331–0.541; *p* < 0.001). Therefore, post-CPB increase in syndecan-1 reflects mostly the preoperative state. It is unclear what causes elevation in syndecan-1 concentration even preoperatively and how it is implicated in the development of AKI postoperatively. As degradation of EG occurs under inflammation, high syndecan-1 concentration may reflect more pro-inflammatory condition. Pesonen and colleagues [22] reported that perioperative syndecan-1 concentration was positively correlated with pro-inflammatory cytokines in patients underwent cardiac surgery with CPB. Likewise, TNF-α concentration was elevated preoperatively in patients with high preoperative syndecan-1 and positively correlated with preoperative syndecan-1 (Supplementary Table S3) in our study, implying more pro-inflammatory condition in these patients. Thus, our results suggest that preoperative inflammatory tone as reflected by increased syndecan-1 conveys significant influence on the degree of ensuing post-CPB inflammation that is associated with dismal prognosis. This association also empowers the role of preoperative syndecan-1 in accurate risk stratification. 

In addition, high preoperative syndecan-1 may reflect baseline endothelial dysfunction. We found that elevated preoperative serum creatinine was more frequent in patients with high preoperative syndecan-1 with a borderline statistical significance. In congruence with our study, Padberg and colleagues [23] demonstrated that plasma syndecan-1 levels increased across chronic kidney disease stages and were independently associated with renal dysfunction. However, the 24-h clearance of syndecan-1 was not associated with that of creatinine, excluding the possibility that elevations in serum syndecan-1 result from reduced glomerular filtration. Furthermore, they confirmed correlations between elevated syndecan-1 levels and several endothelial activation markers. However, in our study, preoperative syndecan-1 predicted severe AKI independently of the preoperative serum creatinine. Thus, the predictive ability of preoperative syndecan-1 may not be explained by preoperatively impaired kidney function alone.

Systemic elevation of syndecan-1 was also observed in other end-organ damage including lung and liver [24]. Because the serum syndecan-1 is not specifically originated from the kidney vasculature, it may represent endothelial dysfunction and microcirculatory disturbance at an end-organ level in general, and AKI can be regarded as an example of frequently developing, accurately measurable end-organ dysfunction after cardiac surgery.

Our results also showed that severe tricuspid regurgitation was more frequent in patients with high preoperative syndecan-1. Reduced renal perfusion pressure due to severe tricuspid regurgitation may contribute to the deterioration of renal function [25]. The causal relationship between increased EG degradation and severe tricuspid regurgitation has not been verified, but it can be speculated that atrial stretch induces the release of atrial natriuretic peptide (ANP), which is known to cause EG degradation [26]. It may also be speculated that venous congestion resulting in increased capillary pressure leads to microcirculatory EG degradation and endothelial dysfunction. The greater right ventricular systolic pressure in patients with high preoperative syndecan-1 suggest more congestion in those patients.

Notably, unlike in previous studies, heparan sulfate concentrations decreased post-CPB from baseline in our study. The reason for this discrepancy is unclear but may be related to the cross-reactivity of protamine to heparan sulfate and exogenous heparin [27], or kinetics of heparan sulfate released into circulation. Heparan sulfate may be rapidly excreted, or sequestered in other organs, for example liver, as shown in liver transplantation resulting in low serum heparan sulfate post-reperfusion [28]. Considering that syndecan-1 contains chondroitin/dermatan sulfate chains as well, measuring these glycosaminoglycans in addition to heparan sulfate might have given us more insights regarding its unexpected decrease after CPB, which remains to be elucidated through a further study.

Fluid resuscitation is an essential part of anesthesiology practice, and EG is thought to play a central role in maintaining the oncotic pressure gradient between intravascular and interstitial space. In our study, the amounts of allogeneic plasma product transfusion and urine output were greater in patients with high preoperative syndecan-1 concentration. The amount of crystalloid fluid administration was also numerically higher in patients with high preoperative syndecan-1. Considering many possible confounding factors, it is hard to relate these observed differences with perioperative EG degradation or increased vascular permeability, which is beyond the scope of the current study. Moreover, preoperative level of syndecan-1 cannot be a measure of degree of perioperative EG degradation.

This study has several limitations. First, it was a single-center study. However, the incidence of AKI was similar to that in previous studies [29,30]. Second, the degree of EG degradation was assessed by measuring serum concentrations of released EG constituents, including syndecan-1 and heparan sulfate. Serum concentrations of EG constituents have been widely used as markers for EG degradation. However, this method is not without drawbacks, as it represents the balance between the rate of degradation and excretion but does not reflect the rate of EG restoration. Furthermore, fold increase or difference between the measurements are disproportionally influenced by the value of initial measurement. Therefore, increased EG constituent concentrations may not accurately represent current EG dimensions or amount of loss, especially in the course of the ongoing processes of degradation and restoration. Third, the method for serum syndecan-1 measurement is not standardized. Therefore, the absolute values presented in this study are not directly applicable to other studies or clinical practice. Finally, this study is not sufficiently powered for multiple subgroup comparisons. Thus, any differences between the low and high syndecan-1 group is not conclusive and should be viewed as exploratory. 

## 5. Conclusions

In conclusion, a high preoperative syndecan-1 concentration greater than 90 ng/mL was an independent predictor of severe AKI after valvular heart surgery, and was associated with increased preoperative inflammatory tone and prolonged postoperative hospitalization.

## Figures and Tables

**Table 1:** Demographic and clinical subject characteristics.

	Total (5–884 ng/mL) (*N* = 250)	Low SDC-1 Group (0–89 ng/mL) (*N* = 191)	High SDC-1 Group (90–884 ng/mL) (*N* = 59)	*p* Value
Age (years)	66 (57–73)	66 (57–73)	65 (56–74)	0.648
Male (*n* (%))	118 (47.2)	87 (45.5)	31 (52.5)	0.347
Body mass index (kg/m^2^)	23.9 ± 3.8	24.0 ± 4.0	23.7 ± 2.9	0.567
Hypertension	133 (53.2)	108 (56.5)	25 (42.4)	0.057
Diabetes mellitus	48 (19.2)	36 (18.8)	12 (20.3)	0.799
Chronic obstructive lung disease	9 (3.6)	6 (3.1)	3 (5.1)	0.690
Preoperative serum Cr >1.2 mg/dL	15 (6.0)	7 (3.7)	8 (13.6)	0.010
Prior myocardial infarction	8 (3.2)	6 (3.1)	2 (3.4)	0.924
Congestive heart failure	55 (22.0)	38 (19.9)	17 (28.8)	0.148
Coronary artery occlusive disease	35 (14.0)	28 (14.7)	7 (11.9)	0.589
Peripheral artery occlusive disease	3 (1.2)	2 (1.0)	1 (1.7)	>0.999
Liver cirrhosis	9 (3.6)	4 (2.1)	5 (8.5)	0.036
Preoperative steroid use	5 (2.0)	5 (2.6)	0 (0.0)	0.344
Preoperative inotrope use	9 (3.6)	5 (2.6)	4 (6.8)	0.221
Severe aortic stenosis	62 (24.8)	47 (24.6)	15 (25.4)	0.899
Severe aortic regurgitation	54 (21.6)	44 (23.0)	10 (16.9)	0.321
Severe mitral stenosis	25 (10.0)	15 (7.9)	10 (16.9)	0.042
Severe mitral regurgitation	75 (30)	65 (34.0)	10 (16.9)	0.012
Severe tricuspid regurgitation	59 (23.6)	34 (17.8)	25 (42.4)	<0.001
**Type of surgery**				0.238
Mitral valve repair/replacement	116 (46.4)	84 (44.0)	32 (54.2)	
Aortic valve replacement	72 (28.8)	62 (32.5)	10 (16.9)	
Double valve surgery	23 (9.2)	16 (8.4)	7 (11.9)	
Valve + CABG	22 (8.8)	16 (8.4)	6 (10.2)	
Valve + aorta	17 (6.8)	13 (6.8)	4 (6.8)	
Left ventricular ejection fraction (%)	62 ± 11	62 ± 12	60 ± 9	0.175
LA volume index (mL/m^2^)	64 (43–94)	59 (39–88)	67 (52–112)	0.067
RV systolic pressure (mmHg)	36 (28–46)	33 (27–43)	41 (33–51)	0.001
Cleveland score	2 (2–3)	2 (1–3)	2 (2–3)	0.088
EuroSCORE	5 (3–7)	5 (3–7)	5 (3–8)	0.371
**Pre-operative medication**				
Beta-blockers	95 (38.0)	70 (36.6)	25 (42.4)	0.429
RAS antagonists	137 (54.8)	109 (57.1)	28 (47.5)	0.195
Calcium-channel blockers	59 (23.6)	51 (26.7)	8 (13.6)	0.053
Antiplatelet agent	57 (22.8)	49 (25.7)	8 (13.6)	0.075
Heparin	77 (30.8)	45 (23.6)	32 (54.2)	<0.001
Diuretics	154 (61.6)	110 (57.6)	44 (74.6)	0.019
Statins	94 (37.6)	75 (39.3)	19 (32.2)	0.328
Digoxin	41 (16.4)	28 (14.7)	13 (22.0)	0.181

Note: Data are expressed as *n* (%), mean ± standard deviation or median (interquartile range). Abbreviations: SDC-1, syndecan-1; Cr, creatinine; CABG, coronary artery bypass graft; LA, left atrium; RV, right ventricle; RAS, renin-angiotensin system.

**Table 2:** Degree of renal injury and changes in renal function.

	Total (*N* = 250)	Low SDC-1 (*N* = 191)	High SDC-1 (*N* = 59)	*p* Value
Acute kidney injury	47 (18.8)	28 (14.7)	19 (32.2)	0.003
Stage 1	34 (13.6)	23 (12.0)	11 (18.6)	
Stage 2	6 (2.4)	2 (1.0)	4 (6.8)	
Stage 3	7 (2.8)	3 (1.6)	4 (6.8)	
Oliguria	7 (2.8)	3 (1.5)	4 (6.8)	0.025
Renal replacement therapy	3 (1.2)	0 (0.0)	3 (5.1)	0.013
Serum creatinine (mg/dL)				
Baseline	0.83 ± 0.23	0.81 ± 0.20	0.92 ± 0.27	0.024
Postoperative 6 h	0.74 ± 0.28	0.71 ± 0.26	0.84 ± 0.32	0.024
Postoperative 24 h	0.97 ± 0.71	0.91 ± 0.75	1.14 ± 0.52	0.048
Postoperative 48 h	0.93 ± 1.21	0.89 ± 1.36	1.05 ± 0.43	0.688

Note: Data are expressed as *n* (%) or mean ± standard deviation. Acute kidney injury, Oliguria and need for renal replacement therapy were assessed during postoperative 48 h. Oliguria was defined as urine output <0.5 mL/kg/h for 6 h. Abbreviation: SDC-1, syndecan-1.

**Table 3:** Intraoperative and 48 h postoperative data.

	Total (*N* = 250)	Low SDC-1 (*N* = 191)	High SDC-1 (*N* = 59)	*p* Value
Duration of CPB (min)	95 (70–120)	90 (70–120)	105 (75–136)	0.131
Crystalloid (mL)	5859 (4784–6982)	5791 (4703–5791)	6406 (5248–7554)	0.010
Colloid (mL)	600 (450–1000)	600 (450–980)	650 (450–1100)	0.260
Patients requiring pRBC transfusion (*n* (%))	148 (59.2)	111 (58.1)	37 (62.7)	0.530
Amount of pRBC transfusion (mL)	280 (0–560)	280 (0–280)	280 (0–560)	0.107
Patients requiring FFP/cryoprecipitate/platelet transfusion (*n* (%))	91 (36.4)	58 (30.3)	33 (55.9)	<0.001
Amount of FFP/cryoprecipitate/platelet transfusion (mL)	0 (0–608)	0 (0–260)	260 (0–1280)	<0.001
Urine output (mL)	6358 (5430–7408)	6175 (5340–7230)	7000 (6165–7650)	0.007
Chest tube drainage (mL)	470 (307–645)	440 (290–617)	532 (360–721)	0.022
Furosemide dose (mg)	55 (25–80)	50 (20–75)	60 (40–100)	0.019
Norepinephrine dose (μg)	972 (424–3329)	882 (416–2875)	1235 (469–3760)	0.339
Vasopressin dose (unit)	1.0 (0–3.3)	0.9 (0–3.2)	1.0 (0–3.7)	0.274
Patients requiring inotrope (*n* (%))	112 (44.8)	80 (41.8)	32 (54.2)	0.100

Note: Data are expressed as *n* (%) or median (interquartile range). Abbreviations: SDC-1, syndecan-1; CPB, cardiopulmonary bypass; pRBC, packed red blood cell; FFP, fresh frozen plasma.

**Table 4:** Inflammatory markers.

	Total (*N* = 250)	Low SDC-1 (*N* = 191)	High SDC-1 (*N* = 59)	*p* Value
TNF-α (pg/mL)				
Post-induction	1.58 (1.17–2.06)	1.45 (1.14–1.92)	1.85 (1.37–2.43)	<0.001
Post-CPB	6.00 (3.77–9.59)	5.92 (3.39–9.61)	6.20 (3.90–9.55)	0.738
IL-6 (pg/mL)				
Post-induction	3.43 (2.18–6.72)	3.10 (2.05–6.28)	4.93 (2.71–9.68)	0.018
Post-CPB	92.1 (26.6–283.7)	84.9 (23.1–247.8)	198.4 (53.1–308.7)	0.062
C-reactive protein (mg/L)				
Preoperative	1.3 (0.6–2.9)	1.2 (0.6–2.6)	1.8 (0.9–5.4)	0.060
Postoperative 6 h	10.3 (2.8–18.2)	9.0 (3.2–19.9)	10.4 (2.5–18.0)	>0.999
Postoperative 24 h	75.9 (52.7–105.5)	75.2 (50.8–98.5)	86.1 (65.5–147.1)	0.040
Postoperative 48 h	164.7 (131.8–204.6)	162.3 (131.0–206.3)	175.6 (135.6–204.0)	>0.999

Note: Data are expressed as mean ± standard deviation or median (interquartile range). Abbreviations: SDC-1, syndecan-1; TNF-α, tumor necrosis factor-α; IL-6, interleukin-6; CPB, cardiopulmonary bypass.

**Table 5:** Postoperative outcomes.

	Total (*N* = 250)	Low SDC-1 (*N* = 191)	High SDC-1 (*N* = 59)	*p* Value
Stroke	2 (0.8)	1 (0.5)	1 (1.7)	0.417
Sternal infection	0 (0.0)	0 (0.0)	0 (0.0)	>0.999
Hemostatic reoperation	5 (2.0)	4 (2.1)	1 (1.7)	>0.999
Mechanical ventilation >24 h	15 (6.0)	8 (4.2)	7 (11.9)	0.053
Mortality	2 (0.8)	2 (1.0)	0 (0.0)	>0.999
Length of ICU stay (day)	3 (2–3)	3 (2–3)	3 (2–4)	0.019
Length of hospital stay (day)	14 (11–18)	13 (11–17)	16 (12–24)	0.001

Note: Data are expressed as *n* (%) or median (interquartile range). Abbreviations: SDC-1, syndecan-1; ICU, intensive care unit.

## Supplementary Materials

The following are available online at https://www.mdpi.com/2077-0383/9/6/1803/s1, Table S1. Hematologic variables, Table S2. Hemodynamic variables, Table S3. Correlations with the preoperative syndecan-1.
