# Supplementary material for: High Preoperative Serum Syndecan-1, a Marker of Endothelial Glycocalyx Degradation, and Severe Acute Kidney Injury after Valvular Heart Surgery
Source: J Clin Med. 2020 Jun 10;9(6):1803. doi: 10.3390/jcm9061803 (PMC7356050; doi:10.3390/jcm9061803)
Supplement: Supplementary file 1 [file jcm-09-01803-s001.pdf]

**Supplementary Table 1.** Hematologic variables.

| | **Low SDC-1 (*N* = 191)** | **High SDC-1 (*N* = 59)** | ***p* Value** |
|---|---|---|---|
| Haematocrit (%) | | | |
| Preoperative | 37.9 ± 5.4 | 36.5 ± 6.3 | 0.568 |
| Postoperative 6 h | 31.6 ± 4.8 | 30.4 ± 4.8 | 0.428 |
| Postoperative 24 h | 30.4 ± 4.6 | 28.9 ± 6.1 | 0.348 |
| Postoperative 48 h | 28.3 ± 4.0 | 27.7 ± 3.9 | >0.999 |
| Leukocyte count (/μL) | | | |
| Preoperative | 6212 ± 1773 | 5228 ± 2061 | 0.004 |
| Postoperative 6 h | 11822 ± 4565 | 10711 ± 4445 | 0.408 |
| Postoperative 24 h | 12572 ± 3730 | 12169 ± 3650 | >0.999 |
| Postoperative 48 h | 11691 ± 3576 | 11362 ± 3352 | >0.999 |
| Platelet count (×$10^3$/μL) | | | |
| Preoperative | 195 ± 63 | 178 ± 64 | 0.004 |
| Postoperative 6 h | 119 ± 35 | 117 ± 37 | 0.492 |
| Postoperative 24 h | 107 ± 37 | 101 ± 31 | 0.924 |
| Postoperative 48 h | 93 ± 33 | 89 ± 31 | >0.999 |

Note: Data are expressed as mean ± standard deviation or median (interquartile range). Abbreviation: SDC-1, syndecan-1.

**Supplementary Table 2.** Hemodynamic variables.

| | **Low SDC-1 (*N* = 159)** | **High SDC-1 (*N* = 91)** | ***p* Value** |
|---|---|---|---|
| Mean arterial pressure (mmHg) | | | |
| Post-induction | 84 ± 15 | 84 ± 16 | >0.999 |
| Post-CPB | 70 ± 10 | 68 ± 13 | 0.640 |
| Postoperative 6 h | 78 ± 10 | 76 ± 9 | 0.320 |
| Postoperative 24 h | 81 ± 10 | 78 ± 9 | 0.196 |
| Heart rate (bpm) | | | |
| Post-induction | 69 ± 13 | 68 ± 13 | >0.999 |
| Post-CPB | 80 ± 10 | 82 ± 10 | >0.999 |
| Postoperative 6 h | 82 ± 10 | 84 ± 14 | >0.999 |
| Postoperative 24 h | 80 ± 10 | 85 ± 14 | 0.128 |
| Central venous pressure (mmHg) | | | |
| Post-induction | 10 ± 3 | 11 ± 4 | 0.068 |
| Post-CPB | 11 ± 3 | 10 ± 2 | 0.068 |
| Postoperative 6 h | 8 ± 3 | 9 ± 3 | 0.312 |
| Postoperative 24 h | 7 ± 3 | 9 ± 3 | 0.060 |
| Mean pulmonary arterial pressure (mmHg) | | | |
| Post-induction | 21 ± 6 | 23 ± 7 | 0.472 |
| Post-CPB | 20 ± 3 | 21 ± 3 | >0.999 |
| Postoperative 6 h | 19 ± 5 | 21 ± 4 | 0.052 |
| Cardiac index (L/min/$m^2$) | | | |
| Post-induction | 2.1 ± 0.5 | 2.2 ± 0.6 | >0.999 |
| Post-CPB | 2.6 ± 0.7 | 2.6 ± 0.6 | >0.999 |
| Postoperative 6 h | 2.9 ± 0.6 | 3.1 ± 0.7 | 0.234 |

Note: Data are expressed as mean ± standard deviation. Abbreviations: SDC-1, syndecan-1; CPB, cardiopulmonary bypass.

**Supplementary Table 3.** Correlations with the preoperative syndecan-1.

| | **Spearman's ϱ** | **95% CI** | ***p* Value** |
|---|---|---|---|
| Age (years) | -0.037 | -0.160-0.088 | 0.567 |
| Body surface area ($m^2$) | 0.018 | -0.106-0.142 | 0.772 |
| Left ventricular EF (%) | -0.214 | -0.330-0.091 | 0.001 |
| LAVI ($mL/m^2$) | 0.267 | 0.084-0.324 | 0.001 |
| RVSP (mmHg) | 0.166 | 0.042-0.285 | 0.008 |
| Preoperative serum Cr (mg/dL) | 0.121 | -0.003-0.242 | 0.055 |
| EuroSCORE | 0.050 | -0.075-0.173 | 0.436 |
| CPB time (min) | 0.249 | 0.127-0.364 | 0.001 |
| Preoperative TNF-$\alpha$ (pg/mL) | 0.226 | 0.103-0.342 | 0.001 |
| Post-CPB TNF-$\alpha$ (pg/mL) | 0.118 | -0.006-0.239 | 0.062 |
| Preoperative IL-6 (pg/mL) | 0.195 | 0.071-0.312 | 0.002 |
| Post-CPB IL-6 (pg/mL) | 0.257 | 0.135-0.371 | 0.001 |

Abbreviations: CI, confidence interval; EF, ejection fraction; LAVI, left atrial volume index; RVSP, right ventricular systolic pressure; Cr, creatinine; CPB, cardiopulmonary bypass; TNF-$\alpha$, tumor necrosis factor-$\alpha$; IL-6, interleukin-6.
